# Awareness, Knowledge, Attitude, and Practice of Teledentistry among Dental Practitioners during COVID-19: A Systematic Review and Meta-Analysis

**DOI:** 10.3390/medicina58010130

**Published:** 2022-01-15

**Authors:** Galvin Sim Siang Lin, Sze Hui Koh, Karyn Zuhuan Ter, Chia Wei Lim, Sharmin Sultana, Wen Wu Tan

**Affiliations:** 1 Department of Dental Materials, Faculty of Dentistry, Asian Institute of Medicine, Science and Technology (AIMST) University, Bedong 08100, Kedah, Malaysia; 2 Puchong Dental Clinic, Ministry of Health Malaysia, Puchong 47100, Selangor, Malaysia; szehui.koh@gmail.com; 3 KK2 Batu Pahat Clinic, Ministry of Health Malaysia, Batu Pahat 83000, Johor, Malaysia; Karyn_ter@icloud.com; 4 Taman Intan Clinic, Ministry of Health Malaysia, Sungai Petani 08000, Kedah, Malaysia; lcwei95@yahoo.com; 5 Department of Orthodontics, Faculty of Dentistry, Manipal University College Malaysia (MUCM), Bukit Baru 75150, Melaka, Malaysia; sharmin.sultana@manipal.edu.my; 6 Department of Dental Public Health, Faculty of Dentistry, Asian Institute of Medicine, Science and Technology (AIMST) University, Bedong 08100, Kedah, Malaysia; tan_wen@aimst.edu.my

**Keywords:** coronavirus, dental care, oral health, pandemic, telehealth

## Abstract

*Background and Objectives*: This systemic review aims to appraise and analyse the awareness, knowledge, attitude, and practice of teledentistry among dental practitioners during COVID-19. *Materials and Methods*: This review was registered in the PROSPERO database (CRD42021283404). Cross-sectional articles on dental practitioners’ perceptions towards teledentistry published between March 2020 and September 2021 were searched in ten online databases (PubMed, Google Scholar, Web of Science, ScienceDirect, Cochrane, EMBASE, SIGLE, EBSCO, LILACS, and Open Grey). The Joanna Briggs Institute critical appraisal tool was employed to analyse the risk of bias (RoB) of each article, whereas the Oxford Centre for Evidence-Based Medicine recommendation tool was used to evaluate the level of evidence. Data were analysed using the DerSimonian–Laird random effect model based on a single-arm approach. *Results*: Six studies were included and demonstrated Level 3 evidence. A single-arm meta-analysis revealed that dental practitioners had a high level of awareness (70.4%) and attitude (72.5%) towards teledentistry during the COVID-19 pandemic, but their knowledge level (57.9%) was moderate with a poor practice level (35.8%). A substantial heterogeneity was observed with the overall I2 ranging from 90.78% to 98.21%. Furthermore, meta-regression indicated that the sample size of each study had a significant (*p* < 0.05) impact on the degree of data heterogeneity. *Conclusions*: Despite their high degree of awareness and attitude, dental practitioners demonstrated moderate knowledge and relatively poor practice of teledentistry during the COVID-19 pandemic. More well-designed studies are warranted to investigate the alternatives for enhancing dental practitioners’ knowledge and practice of teledentistry interventions.

## 1. Introduction

The World Health Organization (WHO) declared the coronavirus disease 2019 (COVID-19) as the first coronavirus pandemic to strike the global healthcare system in March 2020 [1]. Many nations have implemented quarantine and mitigation measures to halt the disease’s transmission. Most ordinary non-emergency healthcare was briefly suspended due to the pandemic’s lockdown, restricting individuals’ contact and access to healthcare concerns, including dental treatment [2,3]. Neglecting oral health issues might result in future dental problems, patient emotional suffering, or even impair their overall quality of life. Dental practitioners are considered high-risk professionals as they are constantly exposed to infections transferred by saliva, blood, or fluid from the nasopharyngeal area due to the nature of dental treatments and close interaction with patients [3,4]. As a result, teledentistry has the potential to give an innovative alternative for continuing dental practice during the pandemic and beyond [2].

Teledentistry, like telemedicine, is the distant or remote delivery of dental care, counselling, education, or treatment using digital technologies rather than physical face-to-face interaction with patients [2]. Telediagnosis, teleconsultation, teletriage, electronic patient records and referrals, and telemonitoring are just a few of the main modalities in modern dentistry practice [2]. Teledentistry has proven to be effective and reliable for distant dental screening, diagnosis, consultation, and treatment planning over the years [1,5]. Moreover, teledentistry is advocated as one of the emergency measures for coping with the COVID-19 pandemic, and a previous study has demonstrated that both dentists and patients feel more secure using teledentistry to minimize non-essential interaction during the pandemic [6]. Since the transition from COVID-19 being a pandemic to an endemic is becoming highly probable, widespread adoption of teledentistry during and after the pandemic is critical, as the primary goal is to avoid face-to-face contact, especially for vulnerable groups, and limit the transmission of this contagious disease [7,8].

Understanding how dentists perceived teledentistry as an alternative tool during the pandemic crisis and how teledentistry may affect future dental professionals’ practice are of paramount importance. Although various surveys on dental practitioners’ perceptions of the employment of teledentistry during the COVID-19 pandemic have been conducted [3,4,7,8], there has yet to be a systematic evaluation and analysis of dental practitioners’ awareness, knowledge, attitude, and practice towards teledentistry. Hence, it is imperative to delve deeper into this context and allow further stakeholders to develop a comprehensive approach for the effective and long-term use of telecommunications for dental care during or even after the pandemic. Thus, the aim of this review is to systematically appraise and analyse the awareness, knowledge, attitude, and practice of teledentistry among dental practitioners during COVID-19.

## 2. Materials and Methods

### 2.1. Protocol and Registration

A study protocol based on the Preferred Reporting Items for Systematic Reviews and Meta-Analyses Protocols (PRISMA) was created [9], and the study was registered with a registration number (CRD42021283404) at the Prospective Register of Systematic Reviews (PROSPERO), University of York.

The focus question was developed based on the POT framework, which includes the Population (P), Outcome of interest (O), and Time (T). The POT criteria were: (1). Population: dental practitioners including general dentists, dental specialists, dental educators or lecturers, and postgraduate dental students (2). Outcome: awareness, knowledge, attitude, and practice on teledentistry (3). Time: during the COVID-19 pandemic.

The POT question was ‘What is the level of awareness, knowledge, attitude and practice among dental practitioners on teledentistry during the COVID-19 pandemic?’ In this context, a dental practitioner is referred to as a dentist who is qualified and certified by the state to provide dental treatments within the extent of their licence and certification. Moreover, the use of information technology and telecommunications for dental treatment, consultation, education, and public awareness is referred to as teledentistry.

### 2.2. Search Strategy

An electronic search was conducted independently by two investigators (GSSL, SHK) using ten electronic databases to identify relevant articles published between March 2020 and September 2021: PubMed, Google Scholar, Web of Science, ScienceDirect, Cochrane, EMBASE, SIGLE, EBSCO, LILACS, and Open Grey. Two other investigators (KZT, CWL) independently evaluated and appraised the reference lists of relevant papers from the electronic search using computer software (EndNote X9, Thomson Reuters). Keywords such as ‘knowledge’, ‘awareness’, ‘attitude’, ‘practice’, ‘teledentistry’, ‘e-dentistry’, ‘COVID-19′, and ‘pandemic’ were applied for each database in conjunction with the use of Boolean operators ‘AND’ and ‘OR’.

### 2.3. Study Selection

After discarding duplicate articles using EndNote software version x9, two investigators (GSSL, SHK) separately screened the titles and abstracts of all the articles, and the remaining two investigators (KZT, CWL) performed a full-text assessment to identify studies based on the inclusion and exclusion criteria. Articles that fulfilled the following inclusion criteria were considered:

- Awareness, knowledge, attitude, and practice towards teledentistry during the COVID-19 pandemic;
- General dentists, dental specialists, dental educators, or postgraduate dental students;
- Cross-sectional study;
- No limit to any country;
- No limit to any language.
- Articles that fulfilled the following exclusion criteria were omitted:
- Expert opinions, reviews, case reports or case series, letters to the editor, short communications
- Studies performed before March 2020 (as the pandemic was announced by WHO in March 2020)
- Full text unavailable

To evaluate interrater reliability, calibrations between investigators were performed. The average concordance was computed using the Kappa value to compare the investigators’ decisions on inclusion and exclusion [10]. Any disagreements that arose throughout the search were addressed and resolved with the fifth investigator (SS).

### 2.4. Data Extraction

Each article’s parameters were extracted and documented by four investigators (GSSL, SHK, KZT, CWL) using customized excel spreadsheet extraction forms: titles of articles, authors, year of publication, country, type of study, sample size, gender, age, assessment method, evaluation criteria, response rate, and overall main results. The fifth investigator (SS) double-checked the accuracy of the filled data and a further discussion with all investigators was conducted if any discrepancies were found.

### 2.5. Risk of Bias Assessment and Level of Evidence

Three investigators (SHK, KZT, CWL) independently assessed the risk of bias (RoB) of the selected articles using the Joanna Briggs Institute (JBI) critical appraisal checklist for analytical cross-sectional studies [11]. Either ‘yes’, ‘no’, ‘unclear’, or ‘not applicable’ was assigned for each domain. Subsequently, the studies were categorized as ‘include’, ‘exclude’, or ‘seek further info’. Any persistent disputes were resolved with the assistance of the fourth investigator (GSSL). The Oxford Centre for Evidence-Based Medicine (OCEBM) guideline was used to establish the level of evidence in each study [12].

### 2.6. Statistical Analysis

The extracted proportions of the awareness, knowledge, attitude, and practice among dental practitioners towards teledentistry during the COVID-19 pandemic from each study were pooled and estimated using single-arm meta-analysis based on the DerSimonian–Laird random-effects model. The analysis was conducted using the OpenMeta (Analyst) software (CEBM, Oxford, UK) with a significance level of 0.05 and 95% confidence intervals (CI). If the estimated upper limit of the 95% confidence interval was larger than 1.0, the upper limit was defined as 1.0. The Higgins’ *I*^2^ statistics were employed to determine the degree of data heterogeneity among the included studies (*I*^2^ less than 30% = acceptable heterogeneity, *I*^2^ between 30 and 60% = moderate heterogeneity, *I*^2^ greater than 60% = substantial heterogeneity) [13]. Subgroup analysis comparing various populations, genders, and age groups with different educational levels was not feasible due to a scarcity of data from the included studies. However, meta-regression was conducted to assess the effect of sample size on the outcomes. Furthermore, Egger’s test was performed to identify publication bias.

## 3. Results

### 3.1. Study Selection

**Figure 1:**
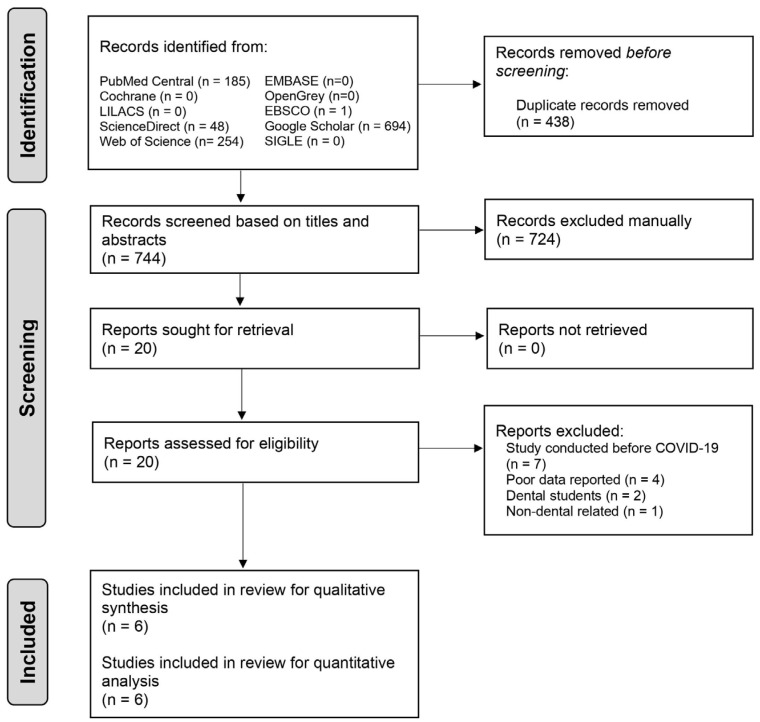
Study selection according to the Preferred Reporting Items for Systematic Reviews and Meta-Analysis (PRISMA) flowchart.

During the initial search, a total of 1182 articles were retrieved (Figure 1). A total of 438 articles were eliminated after duplication was removed, followed by 724 articles being discarded after screening based on titles and abstracts; the remaining 20 articles were chosen for full-text evaluation. Finally, only six studies were included in the current review encompassing a total of 6904 dental practitioners. The average inter-investigators’ Kappa score was 0.82 during the study selection process, which indicates a ‘perfect’ agreement [14]. Figure 1 depicts the reasons for article exclusion.

### 3.2. Study Characteristics

Table 1 summarized the characteristics of each included study. All the included articles were cross-sectional studies. Specific survey questions from each included article were extracted to represent the awareness, knowledge, attitude, and practice towards teledentistry. Five studies focused on the awareness of teledentistry among dental practitioners during the COVID-19 pandemic [3,4,7,8,15], five studies on their knowledge [4,7,8,15,16], four studies on their attitude [3,4,7,8], and three studies on their practice of teledentistry [3,8,15]. Among all the included studies, three articles originated from Pakistan [4,7,15], and one article each from Colombia [3], Saudi Arabia [8], and India [16].

### 3.3. Risk of Bias Assessment and Level of Evidence

All included studies in the present review were deemed ‘include’ based on the JBI critical appraisal tool (Table 2). All the included studies were rated ‘Yes’ for domains 1, 2, 3, 4, 5, 7, and 8, but one study was rated ‘No’ for domain 6 [4]. All the included studies were ranked as Level 3 based on the evidence of OCEBM. The risk of bias assessment and level of evidence’s Cohen’s kappa coefficient (κ) were scored 0.78 and 0.80, respectively, indicating a ‘substantial’ agreement [14].

### 3.4. Statistical Analysis

**Figure 2:**
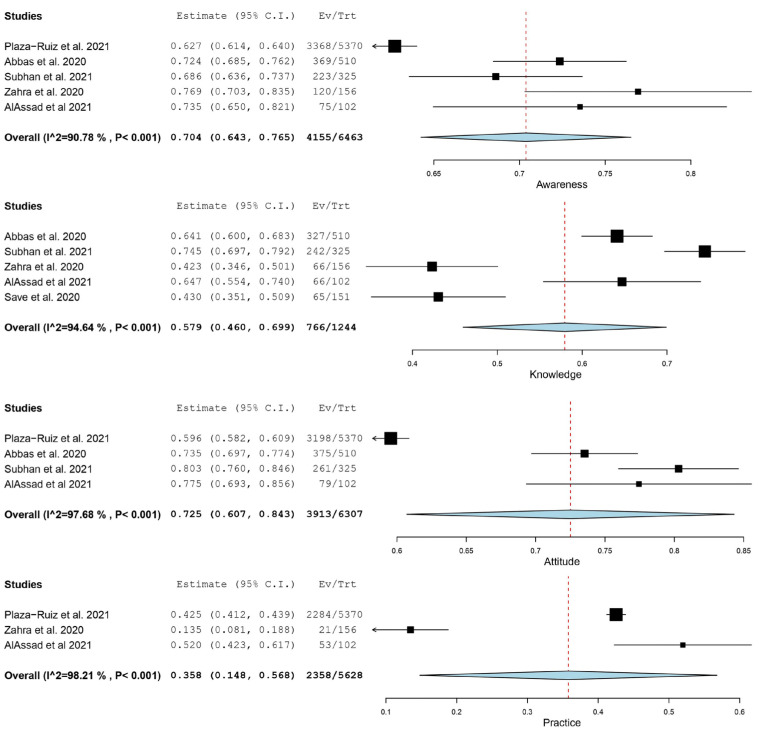
Single-arm meta-analysis showing the awareness, knowledge, attitude, and practice level of teledentistry among dental practitioners.

Table 3 shows the proportions of the level of awareness, knowledge, attitude, and practice towards teledentistry among dental practitioners during the COVID-19 pandemic retrieved from the included studies. Quantitative syntheses were performed when three or more studies were available for each evaluation criteria. For each study, participants such as general dentists, dental specialists, postgraduate dental students and dental educators or lecturers were pooled together. Based on the single-arm meta-analysis (Figure 2), a high level of awareness (70.4%, CI: (64.3, 76.5)) and attitude (72.5%, CI: (60.7, 84.3)) towards teledentistry was noted among dental practitioners during the COVID-19 pandemic. However, the knowledge level (57.9%, CI: (46.0, 69.9)) were deemed moderate, while their practice level of teledentistry (35.8%, CI: (14.8, 56.8)) was found to be poor among dental practitioners. Overall, the *I*^2^ of the weighted mean awareness, knowledge, attitude, and practice level of teledentistry among dental practitioners ranged from 90.78% to 98.21%, indicating a substantial degree of data heterogeneity (*p* < 0.001) among the studies included.

Sensitivity analyses were carried out by removing each data set one at a time. The highest and lowest weight mean awareness levels were 72.3% (CI: 69.1, 75.5) and 68.8% (CI: 62.7, 74.9), when Plaza-Ruiz et al. [3] and Zahra et al. [15] were omitted, respectively. Furthermore, the highest and lowest weight mean knowledge levels were 61.8% (CI: 50.3, 73.4) and 53.6% (CI: 41.0, 66.3), when Zahra et al. [15] and Subhan et al. [4] were removed, respectively. The highest and lowest weight mean attitude levels were 76.9% (CI: 72.2, 81.7) and 69.8% (CI: 57.9, 81.7), when Plaza-Ruiz et al. [3] and Subhan et al. [4] were eliminated, respectively. Finally, the highest and lowest weight mean practice levels were 46.0% (CI: 37.1, 54.9) and 32.5% (CI: 19.2, 70.2), when Zahra et al. [15] and Plaza-Ruiz et al. [3] were omitted, respectively.

Meta-regression analysis (Appendix A) was also performed to assess the effect of the participants’ sample size of each study on the degree of awareness, knowledge, attitude, and practice towards teledentistry. Significant differences were found for all evaluating criteria (*p*-values: awareness (<0.001), knowledge (<0.001), attitude (<0.001), and practice (0.004)) signifying that the sample size of each study had a direct effect on the degree of data heterogeneity. In addition, Egger’s test revealed that there was no indication of significant publication bias in the level of awareness, knowledge, attitude, and practice of teledentistry among dental practitioners during the COVID-19 pandemic (Egger’s test: *p*-value = 0.32, 0.021, 0.05, and 0.11, respectively).

## 4. Discussion

The current review is the first of its kind to comprehensively evaluate the perceptions of dental practitioners including their awareness, knowledge, attitude, and practice towards teledentistry during the COVID-19 pandemic. Teledentistry enables distance communication and consultation by avoiding face-to-face contact and allowing the exchange of clinical information [2,15]. It also facilitates remote oral care and patient education, which are recommended by healthcare authorities around the globe, particularly during the COVID-19 pandemic, when social distancing should be emphasized to prevent the spread of the coronavirus [2,7]. Despite the current review only covering a small number of relevant primary papers, it uncovered valuable insights regarding teledentistry application among dental practitioners.

In the present single-arm meta-analyses, dental practitioners exhibited a high degree of awareness (70.4%) and attitude (72.5%) towards teledentistry. Such a finding corroborates the findings of a previous systematic review on telehealth in which a high level of awareness and attitude were observed among healthcare professionals [17]. Most dental practitioners agreed that teledentistry is a brilliant invention that may bring certain benefits and requires forward thinking, which has resulted in a more favourable attitude towards teledentistry [7,8]. However, the knowledge level among dental practitioners in the present analysis was somewhat moderate (57.9%), which contradicts other similar studies [18,19,20]. This could be due to income, legislation, previous undergraduate education, and infrastructural variations that exist between countries [3]. The current finding suggested that dental practitioners are aware of teledentistry and have a favourable attitude toward it, but they are unclear of the knowledge and skills required to utilise it. One probable explanation is that dental practitioners are not well-exposed to teledentistry through workshops, lectures, or seminars [8]. In addition, work experience, postgraduate qualification, and internet access were found to be major predictors of teledentistry knowledge among dental practitioners. It was also reported that junior dental practitioners and those with a postgraduate degree showed a better level of knowledge towards teledentistry [15,21]. This could be due to the fact that teledentistry is a relatively new concept, and senior dental practitioners may not have received sufficient training to cutting-edge technology. Similarly, dental practitioners with postgraduate qualifications may have had more exposure to IT technology throughout their postgraduate studies.

On the other hand, the practice of teledentistry was still found to be uncommon during the COVID-19 pandemic, with only about 35.8% of dental practitioners using it. It is not surprising that teledentistry practice is still limited, despite their adequate knowledge of the subject [18,22]. Dental practitioners’ knowledge and comprehension of teledentistry, the skills necessary for its effective application, and a working environment favourable to the adoption of such a new technology are all critical attributes in the widespread acceptance and practice of teledentistry [15,16,23]. Inadequate financial remuneration and disparities in rural regions have also been cited as having a detrimental impact on teledentistry application [3]. However, dental practitioners’ knowledge and attitude level towards teledentistry improved during the pandemic period, implying that the increased familiarity and practice of teledentistry would likely continue even when the pandemic entered the endemic phase [3,8].

Another factor that may contribute to a lower level of practice among dental practitioners is that most of the primary studies included in the present systematic review originated from developing countries [4,7,15,16]. While developed countries continue to benefit and expand this technology by encouraging remote health consultation and monitoring with efficient online record-keeping systems, telehealth including telemedicine and teledentistry in developing countries is still in its infancy [23]. Thus, one may postulate that many developing countries still encounter a lack of teledentistry services, and a scarcity of skilled dental practitioners incorporated this technology into their daily practice, making it more challenging to offer remote oral healthcare services during the COVID-19 era, particularly in suburban and remote regions [16]. Despite these issues, the authors believe that the COVID-19 pandemic will provide an excellent opportunity for developing countries to optimise teledentistry by providing greater skills and new technologies that could change the future of dentistry.

Telehealth modalities, such as teledentistry, provide a wealth of advantages, including ease of application, a tendency to enhance outcomes and communication, low cost, the ability to reduce travel time, expand access to treatment, and raise patient self-awareness [24,25]. In light of the present COVID-19 situation and efforts to expand the number of patients treated via teledentistry as a means of limiting virus transmission, dental practitioners may be anticipated to incorporate teledentistry into their work practices on a larger scale. Increased patient acceptance and self-management will likely lead to teledentistry being a more integral element of the care pathway for a variety of oral health issues [2]. Dental practitioners and other dental auxiliaries participating in service design and equipment selection will also assist in boosting teledentistry adoption [26]. Training and continuing professional education can help to enhance teledentistry awareness and knowledge, as well as ensure that dental practitioners are prepared to use it in the treatment pathway [15]. Rather than being viewed as a threat to professional identity and competence, understanding how teledentistry might enable them to accomplish some regular consultation and monitoring activities remotely is essential.

Other factors such as the dental practitioners’ age and gender, their work environment, and educational level may have an impact on the overall results [3,8,19]. These characteristics were not evaluated in the current review because the data obtained from the primary studies were pooled together, making it impossible to split the results into numerous age groups or genders for comparison. Notwithstanding this, meta-regression was employed and discovered that different sample sizes had a considerable impact on the findings. It is not odd that this occurred since the included studies contained a large range of sample size, which might increase the likelihood to skew the results in one direction. Furthermore, the inclusion and assessment criteria vary significantly among the studies because different studies define the terms ‘knowledge’ and ‘awareness’ differently. In the current review, knowledge refers to a profound comprehension and acquaintance of teledentistry, whereas awareness refers to a superficial understanding.

Most of the included studies were considered to have a low risk of bias in all domains except for one study rated ‘No’ for domain 6: ‘Were strategies to deal with confounding factors stated?’ [4]. Subhan R et al. [27] identified cofounding factors including age and gender but did not specify how these factors may affect their findings. Additionally, the recent meta-analyses revealed significant heterogeneity. This might be due to the inclusion of studies with a wide range of sample sizes, as well as the nature of each study’s presentation of all evidence using different forms of questionnaires. Unfortunately, due to the small number of studies, subgroup analysis was not possible. When individual participant’s data are accessible, the sources of heterogeneity and bias may be fully explored, but most included studies only disclosed aggregate data [28].

The present review provides useful information that paves the way for teledentistry by suggesting the creation of more related programmes and software to fill in the gaps between dental practitioners and patients. Healthcare providers and policymakers are advocated to embrace teledentistry and assist legislation in keeping up with the technology by allowing more funding and infrastructural options for teledentistry. Nonetheless, it is worth noting that the confidentiality of patients’ information may be a concern that compromises privacy [4]. Patients’ privacy and the establishment of secure information technology networks should be prioritised when contemplating teledentistry adoption [22].

One drawback of the present study is that the included primary studies were still limited in their ability to generalise and extrapolate the findings of the context into a larger population. To ensure accurate inferential outcomes, a substantial amount of primary research should be included in the meta-analysis, but it is understandable that such a criterion is rarely met, particularly in the field of dentistry where the number of selected studies is often very limited [29]. In addition, the absence of subgroup analysis on the impact of dental practitioners’ age, gender, working environment, and qualifications due to a scarcity of data may have hampered the current review from developing a greater understanding of teledentistry. Sampling and response bias of each primary study was not addressed in the current review as it was beyond our scope. Thus, more well-designed studies from different countries are warranted to obtain a more general understanding of the level of awareness, knowledge, attitude, and practice of teledentistry among dental practitioners.

## 5. Conclusions

The present review findings suggested that a high degree of awareness and attitude toward teledentistry was noted among dental practitioners during the COVID-19 pandemic. On the other hand, their knowledge level was moderate, while practice level was relatively poor. Teledentistry offers the promise to provide a new strategy for continuing dental care during and after the pandemic. Hence, it is imperative that future well-designed studies are warranted to investigate alternative approaches to enhance dental practitioners’ knowledge and practice of teledentistry. The authors also advocate that future studies should further evaluate participants’ characteristics for better comparisons and a deeper understanding of dental practitioners’ perceptions towards teledentistry.

## Figures and Tables

**Table 1:** Characteristics of the included studies.

Study	Year	Country	Study Design	Sample Size	Gender	Mean Age	Evaluation Tool	Evaluation Criteria	Response Rate	Main Result
Plaza-Ruiz et al. [3]	2021	Colombia	cross-sectional	5370 (2252 GD, 2589 DS)	F: 3878, M: 1492	45	questionnaire	awareness, attitude, practice	16.84%	Knowledge and practice of teledentistry increased since the emerging of COVID-19.
Abbas et al. [7]	2020	Pakistan	cross-sectional	510 (GD, PGDS, DE, DS)	n/a	n/a	questionnaire	knowledge, awareness, attitude	100%	Awareness regarding teledentistry is high among general dentists.
Subhan et al. [4]	2021	Pakistan	cross-sectional	350 (GS, DS)	F: 151, M: 174	n/a	questionnaire	awareness, knowledge, attitude	92.80%	Most of the dental professionals had inadequate knowledge about teledentistry before COVID-19, but their awareness and perception were currently satisfactory.
Zahra et al. [15]	2020	Pakistan	cross-sectional	172 (GD, DS, PGDS)	F: 95, M: 61	n/a	questionnaire	knowledge, awareness, practice	90.62%	In total, 76.6% participants had knowledge of teledentistry, but 80.8% had never used it.
AlAssad et al. [8]	2021	Saudi Arabia	cross-sectional	102 (PGDS, GD)	F: 39, M: 63	n/a	questionnaire	knowledge, awareness, attitudes, and practices	78.50%	Adequate knowledge and awareness of teledentistry during the COVID-19 pandemic.
Save et al. [16]	2020	India	cross-sectional	151 (GD, DS)	F: 99, M: 52	25.72	questionnaire	knowledge	100%	Only 43% of the participants were aware of teledentistry.

DE: Dental educators; DS: Dental specialists; GD: General dentists; PGDS: Postgraduate dental students; F: Female; M: Male; n/a: Not Applicable.

**Table 2:** Risk of bias and level of evidence of each included study.

Study	Domains								Overall Appraisal	LOE
	1	2	3	4	5	6	7	8		
Plaza-Ruiz et al. [3]	Y	Y	Y	Y	Y	Y	Y	Y	Include	3
Abbas et al. [7]	Y	Y	Y	Y	Y	Y	Y	Y	Include	3
Subhan et al. [4]	Y	Y	Y	Y	Y	N	Y	Y	Include	3
Zahra et al. [15]	Y	Y	Y	Y	Y	Y	Y	Y	Include	3
AlAssad et al. [8]	Y	Y	Y	Y	Y	Y	Y	Y	Include	3
Save et al. [16]	Y	Y	Y	Y	Y	Y	Y	Y	Include	3

Y: Yes; U: Unclear; N: No; LOE: Level of evidence. Domain 1: Were the criteria for inclusion in the sample clearly defined? Domain 2: Were the study subjects and the setting described in detail? Domain 3: Was the exposure measured in a valid and reliable way? Domain 4: Were objective, standard criteria used for measurement of the condition? Domain 5: Were confounding factors identified? Domain 6: Were strategies to deal with confounding factors stated? Domain 7: Were the outcomes measured in a valid and reliable way? Domain 8: Was appropriate statistical analysis used?

**Table 3:** Awareness, knowledge, attitude, and practice among dental professionals towards teledentistry during COVID-19.

Study	Year	Participants	Awareness	Knowledge	Attitude	Practice
Plaza-Ruiz et al. [3]	2021	GD, DS	(3368/5370)	n/a	(3198/5370)	(2284/5370)
Abbas et al. [7]	2020	GD, PGDS, DE, DS	(369/510)	(327/510)	(375/510)	n/a
Subhan et al. [4]	2021	GD, DS	(223/325)	(242/325)	(261/325)	n/a
Zahra et al. [15]	2020	GD, DS, PGDS	(120/156)	(66/156)	n/a	(21/156)
AlAssad et al. [8]	2021	PGDS, GD	(75/102)	(66/102)	(79/102)	(53/102)
Save et al. [16]	2020	GD, DS	n/a	(65/151)	n/a	n/a

DE: Dental educators; DS: Dental specialists; GD: General dentists; PGDS: Postgraduate dental students. n/a: Not Applicable.

## Data Availability

Not applicable.

## Appendix A

Meta-regression evaluating the effect of sample size of each study on the degree of awareness, knowledge, attitude, and practice toward teledentistry. * Significance at 0.05.

**Categories**	**Coefficient**	**Confidence Intervals**		**Standard Error**	***p*-Value**
		**Upper Bound**	**Lower Bound**		
Awareness	0.729	0.700	0.757	0.015	0.001 *
Knowledge	0.533	0.321	0.745	0.108	0.001 *
Attitude	0.771	0.743	0.798	0.014	0.001 *
Practice	0.319	0.098	0.540	0.113	0.004 *
